# Elevated sperm DNA fragmentation is associated with embryonic developmental attrition after fertilization in conventional IVF cycles

**DOI:** 10.3389/fcell.2026.1925444

**Published:** 2026-09-21

**Authors:** Lijun Peng, Mengyi Zhu, Tengfei Wang, Sheng Yang, Yun Le

**Affiliations:** Department of Assisted Reproduction, Huzhou Maternity & Child Health Care Hospital, Huzhou, Zhejiang, China

**Keywords:** blastocyst formation, conventional IVF, day-3 embryo quality, DNA fragmentation index, embryonic developmental attrition, paternal DNA integrity, post-fertilization development, sperm DNA fragmentation

## Abstract

**Background:**

The influence of sperm DNA fragmentation on conventional *in vitro* fertilization (IVF) outcomes remains controversial, particularly regarding whether its embryological relevance is expressed during fertilization or subsequent embryo development. This study aimed to evaluate the association between sperm DNA fragmentation index (DFI) and stage-specific embryological transition outcomes in a retrospective cohort of first eligible conventional IVF cycles.

**Methods:**

This retrospective cohort study included 1,423 first eligible conventional IVF cycles selected from fresh oocyte-retrieval treatment cycles recorded between August 2021 and December 2025. Cycles involving intracytoplasmic sperm injection, rescue intracytoplasmic sperm injection, or mixed fertilization procedures were excluded. Sperm DFI was categorized using a primary threshold of 20%, with cycles classified as lower DFI (≤20%) or elevated DFI (>20%). Embryological development was assessed using stage-specific transition outcomes, including oocyte-to- two-pronuclear (2 P N) formation, 2 P N-to-day-3 good-quality embryo formation, blastocyst formation among embryos cultured for blastocyst development, and blastocyst-to-good-quality blastocyst formation. Binomial regression models were used to estimate odds ratios (ORs) and 95% confidence intervals (CIs). Multivariable models were adjusted for female age, male age, female body mass index, and basal follicle-stimulating hormone. Additional analyses examined clinically interpretable DFI categories and alternative DFI thresholds.

**Results:**

Among the 1,423 cycles, 1,218 were classified as lower DFI and 205 as elevated DFI. Elevated DFI was not associated with the oocyte-to-2PN transition after adjustment (63.7% vs. 62.3%; adjusted OR, 0.96; 95% CI, 0.84–1.10; P = 0.523). In contrast, elevated DFI was associated with a lower probability of 2 P N-to-day-3 good-quality embryo formation (34.5% vs. 29.4%; adjusted OR, 0.71; 95% CI, 0.57–0.87; P = 0.001). Elevated DFI was also associated with reduced blastocyst formation among embryos cultured for blastocyst development (57.3% vs. 50.4%; adjusted OR, 0.80; 95% CI, 0.66–0.96; P = 0.018). Good-quality blastocyst formation among formed blastocysts was 34.4% in the lower-DFI group and 30.8% in the elevated-DFI group (adjusted OR, 0.87; 95% CI, 0.68–1.11; P = 0.267). Analyses using clinically interpretable DFI categories and alternative thresholds showed that the association was most consistently observed for post-fertilization developmental outcomes rather than normal fertilization.

**Conclusion:**

In this retrospective cohort of first eligible conventional IVF cycles, elevated sperm DFI was not independently associated with normal fertilization but was associated with modestly reduced post-fertilization embryological development, particularly 2 P N-to-day-3 good-quality embryo formation and blastocyst formation among embryos cultured for blastocyst development. These findings support a stage-specific developmental framework for evaluating the embryological relevance of paternal DNA integrity in conventional IVF, while suggesting that sperm DFI should not be interpreted as a stand-alone marker of fertilization potential.

## 1 Introduction

Infertility is a common reproductive disorder and remains an important public health concern worldwide. Assisted reproductive technology (ART) has become an established treatment for couples with infertility, and conventional IVF remains widely used when fertilization can be achieved without micromanipulation. Although female age, ovarian reserve, oocyte yield, and embryo morphology are routinely considered in IVF counseling and laboratory evaluation, the contribution of paternal factors to early embryonic development has received increasing attention (Devi et al., 2026; Naz and Kamal, 2017; Wang et al., 2022; Schlegel et al., 2021). Conventional semen analysis provides useful information regarding sperm concentration, motility, and morphology, but these parameters do not fully capture sperm nuclear integrity or the functional competence of spermatozoa during fertilization and subsequent embryonic development (Wang et al., 2022; Schlegel et al., 2021; Raheem and Walsh, 2017).

Sperm DNA fragmentation has emerged as a potential marker of sperm nuclear quality. Increased sperm DNA fragmentation index has been associated with oxidative stress, impaired chromatin packaging, abnormal sperm maturation, and reduced reproductive potential (Esteves et al., 2021). However, the clinical significance of sperm DNA fragmentation in ART remains incompletely defined. Several systematic reviews and meta-analyses have suggested that elevated sperm DNA damage may be associated with poorer pregnancy outcomes, including lower pregnancy or live birth rates and higher pregnancy loss after IVF or intracytoplasmic sperm injection (ICSI) (Simon et al., 2017; Qu et al., 2025; Braga et al., 2023; Zini et al., 2011). In contrast, other studies have reported inconsistent or weak associations between sperm DNA fragmentation and fertilization, embryo quality, or clinical pregnancy, partly because of heterogeneity in study populations, fertilization methods, sperm DNA fragmentation assays, clinical thresholds, and outcome definitions (Zini et al., 2008; Santi et al., 2018; Cissen et al., 2016). Therefore, it remains important to clarify the specific developmental stage at which sperm DNA fragmentation may be most relevant.

From a biological perspective, paternal DNA damage may not necessarily impair fertilization itself. Early events after fertilization are largely supported by maternal transcripts and oocyte-derived repair mechanisms, whereas the paternal genome may become increasingly relevant during later cleavage-stage development and embryonic genome activation (Vassena et al., 2011). Previous work has proposed a “late paternal effect” in which sperm DNA damage may be associated with impaired embryo development after the earliest post-fertilization stages rather than with abnormal zygote formation. This concept is consistent with observations that human embryonic gene expression becomes increasingly active during cleavage-stage development and that paternal genome integrity may contribute to successful progression toward high-quality cleavage-stage embryos and blastocysts (Yuan et al., 2023; Simon et al., 2014). These biological considerations support the value of evaluating sperm DNA fragmentation across defined embryological milestones.

Most previous clinical studies have assessed sperm DNA fragmentation using broad endpoints such as fertilization rate, embryo quality, clinical pregnancy, miscarriage, or live birth. However, IVF is a multistep biological process, and a single summary endpoint may obscure stage-specific developmental attrition. In particular, it remains uncertain whether elevated sperm DFI is mainly associated with impaired oocyte-to-2PN fertilization, reduced development to good-quality day-3 embryos, or compromised blastocyst formation. Moreover, blastocyst outcomes require careful interpretation because embryo allocation to extended culture may be influenced by clinical and laboratory decisions regarding embryo transfer, cryopreservation, and continued culture. A stage-specific analytical framework that clearly defines the denominator for each developmental transition may therefore provide a more precise and transparent understanding of the embryological relevance of paternal DNA integrity in conventional IVF.

Therefore, this retrospective cohort study aimed to investigate the association between sperm DFI and embryological development in a retrospective cohort of first eligible conventional IVF cycles. Embryological development was analyzed as a series of defined transition outcomes, including oocyte-to-2PN formation, 2 P N-to-day-3 good-quality embryo formation, blastocyst formation among embryos cultured for blastocyst development, and blastocyst-to-good-quality blastocyst formation. The primary analysis compared cycles with lower DFI and elevated DFI using a 20% threshold, and additional analyses were performed using clinically interpretable DFI categories and alternative thresholds. By applying stage-specific denominators across successive embryological transitions, this study sought to localize where the association with elevated sperm DFI becomes apparent in conventional IVF and to distinguish fertilization competence from subsequent embryonic developmental competence. The contribution of this approach lies in localizing the association with sperm DFI across successive embryological transitions within the same conventional-IVF cohort, rather than treating fertilization and subsequent embryo development as interchangeable IVF outcomes.

## 2 Materials and methods

### 2.1 Study design and participants

This retrospective cohort study was conducted at the Reproductive Center of Huzhou Maternity and Child Healthcare Hospital. The study used routine semen examination records and reproductive laboratory data from fresh oocyte-retrieval treatment cycles recorded in the center’s cycle-search database between August 2021 and December 2025. A fresh cycle was defined by the oocyte-retrieval treatment cycle and did not require fresh embryo transfer. All data were analyzed after de-identification.

The primary analysis was restricted to the first eligible conventional IVF cycle per couple. Cycles were eligible if sperm DFI was available and core embryological records could be traced, including the number of oocytes undergoing conventional IVF insemination, normally fertilized 2 P N embryos, cleaved embryos, day-3 good-quality embryos, embryos cultured for blastocyst formation, blastocysts formed, and good-quality blastocysts when applicable.

To maintain a homogeneous conventional IVF cohort, cycles were excluded if intracytoplasmic sperm injection, rescue intracytoplasmic sperm injection, or mixed IVF and ICSI insemination was performed. Cycles were also excluded if key DFI or embryological count data were missing or internally inconsistent. When more than one eligible cycle was identified for the same female patient, only the first eligible cycle was retained.

A total of 2,429 fresh oocyte-retrieval treatment-cycle records were identified from the cycle-search database. After sequential filtering, 2,427 fresh IVF oocyte-retrieval treatment cycles, 2064 cycles labeled as conventional IVF, 1872 cycles without ICSI or rescue ICSI counts, and 1,665 cycles with valid sperm DFI and core 2 P N-based embryological records remained. After retaining only the first eligible conventional IVF cycle per female patient, 1,423 cycles were included in the final analytical cohort.

The study protocol was approved by the Institutional Review Board of Huzhou Maternity and Child Healthcare Hospital (No. 2025-J-028). Owing to the retrospective nature of the study and the use of de-identified routine laboratory data, the requirement for written informed consent was waived.

### 2.2 Semen assessment and sperm DNA fragmentation testing

Routine semen parameters, including sperm concentration, motility, and morphology, were assessed according to the laboratory standard operating procedures based on the World Health Organization laboratory manual. Detailed procedures for routine semen assessment are provided in the Supplementary Material.

Sperm DNA fragmentation was assessed using an acridine orange–based sperm nuclear integrity fluorescence assay (Sperm Nuclear Integrity Staining Solution, fluorescence staining method; Tianjin Meiruite Medical Technology Co., Ltd., Tianjin, China). The commercial kit comprised Reagent A (sperm staining solution), Reagent B (acid-treatment solution), and Reagent C (sperm diluent). According to the manufacturer’s protocol, liquefied semen was diluted with Reagent C to approximately 1 × 10^6^ sperm/mL. A 200-μL aliquot of the diluted sample was mixed with 400 μL of Reagent B. After 30 s of acid treatment, 1.0 mL of Reagent A was added, followed by incubation for 5 min protected from light. Flow-cytometric analysis was then performed using a BriCyte E6 flow cytometer (Mindray, Shenzhen, China).

The assay is based on differential acridine-orange fluorescence after acid-induced DNA denaturation. Tightly condensed native double-stranded sperm DNA predominantly exhibits green fluorescence, whereas DNA susceptible to acid-induced denaturation assumes a predominantly single-stranded state and exhibits increased red fluorescence. A predefined gating template was applied consistently across samples. DFI was determined automatically by the flow-cytometry analysis software and reported as the percentage of sperm events falling within the predefined DNA-fragmented region among the total gated sperm events.

In the present study, sperm DFI was categorized using a primary analytical threshold of 20% to define lower DFI (≤20%) and elevated DFI (>20%). The 20% threshold was specified before outcome modeling to provide a prespecified analytical contrast between cycles with relatively lower and elevated sperm DNA fragmentation while retaining sufficient observations in the elevated-DFI group for stage-specific analyses. Because DFI thresholds are assay- and study-dependent, additional analyses were performed using DFI categories of ≤15%, 15%–20%, 20%–30%, and >30%, together with alternative thresholds of 15%, 25%, and 30%, to examine whether the observed associations depended on the primary 20% cutoff.

### 2.3 IVF laboratory procedures and embryo assessment

All cycles included in the analytical cohort underwent conventional IVF insemination. Fertilization was assessed 18–20 h after insemination by examination of pronuclei and polar bodies, and normal fertilization was defined as the presence of two pronuclei and two polar bodies.

Cleavage-stage embryos were evaluated according to blastomere number, symmetry, cytoplasmic appearance, and fragmentation. Day-3 good-quality embryos were defined as grade I or II embryos with developmentally appropriate cleavage according to the laboratory grading system. The designation “day-3 good-quality embryo” refers to morphological assessment on day 3 of culture and is independent of the subsequent timing of embryo transfer.

Blastocysts were evaluated according to the Gardner morphological grading system using expansion stage, inner cell mass morphology, and trophectoderm morphology. Good-quality blastocysts were defined as day-5 blastocysts graded 3 B B or higher and day-6 blastocysts graded 4 B B or higher. Detailed ovarian stimulation, embryo-culture, and morphological grading procedures are provided in the Supplementary Material.

### 2.4 Outcome definitions

The embryological outcomes were selected to distinguish normal fertilization from subsequent stages of embryo development while preserving the appropriate denominator for each developmental transition. Oocyte-to-2PN formation was selected to assess normal fertilization after conventional IVF. The primary outcome, 2 P N-to-day-3 good-quality embryo formation, was selected to characterize early post-fertilization developmental competence among normally fertilized embryos and is assessed before subsequent allocation to extended blastocyst culture. Blastocyst formation among embryos entering extended culture was included as a secondary outcome to evaluate later preimplantation development. Good-quality blastocyst formation among formed blastocysts was evaluated as an additional conditional outcome of blastocyst morphology.

Blastocyst formation was treated as a conditional transition outcome because blastocyst status was observable only for embryos entering extended culture. Embryos not undergoing extended culture were not classified as blastocyst failures. The resulting estimand therefore represents blastocyst formation among embryos actually cultured for blastocyst development and is distinct from a 2 P N-based or oocyte-based blastocyst yield.

In this study, a transition rate was defined as the proportion of oocytes or embryos progressing from a specified starting stage to a defined subsequent developmental milestone, using the corresponding stage-specific denominator. Oocyte-to-2PN formation was defined as the number of normally fertilized 2 P N embryos divided by the number of oocytes undergoing conventional IVF insemination. 2 P N-to-day-3 good-quality embryo formation was defined as the number of day-3 good-quality embryos divided by the number of normally fertilized 2 P N embryos. Blastocyst formation was defined as the number of blastocysts formed divided by the number of embryos cultured for blastocyst formation. Good-quality blastocyst formation was defined as the number of good-quality blastocysts divided by the number of blastocysts formed.

These outcomes were analyzed separately rather than as a single continuous cascade because each transition had its own laboratory denominator. This approach was used to avoid mixing oocyte-based, 2 P N-based, culture-based, and blastocyst-based denominators in the same calculation. Cycles with valid fertilization data were retained in the analytical cohort; cycles with zero 2 P N embryos contributed to the oocyte-to-2PN analysis but were not informative for the 2 P N-to-day-3 transition model.

Pregnancy and live-birth outcomes were not study endpoints because the present analysis focused on stage-specific embryological development within the oocyte-retrieval cycle. Evaluation of downstream reproductive outcomes at the retrieval-cycle level would require cumulative linkage of all fresh and subsequent frozen-thawed embryo transfers arising from the same retrieval cycle.

### 2.5 Statistical analysis

Continuous variables were summarized as median with interquartile range, and categorical variables were summarized as counts and percentages. Baseline characteristics were compared between the lower DFI and elevated DFI groups using the Mann Whitney U test for continuous variables and the chi square test or Fisher’s exact test for categorical variables, as appropriate.

Embryological outcomes were analyzed as binomial transition outcomes using the corresponding numerator and denominator for each developmental stage. The primary exposure was elevated sperm DFI, defined as DFI >20%, with DFI ≤20% used as the reference group. The four transition outcomes were oocyte-to-2PN formation, 2 P N-to-day-3 good-quality embryo formation, blastocyst formation among embryos cultured for blastocyst development, and blastocyst-to-good-quality blastocyst formation.

Binomial regression models were used to estimate odds ratios and 95% confidence intervals for the association between elevated sperm DFI and each embryological transition outcome. Unadjusted and multivariable adjusted models were fitted. Multivariable models were adjusted for female age, male age, female body mass index, and basal follicle stimulating hormone. These covariates were selected *a priori* because of their potential associations with ovarian response, embryo development, and IVF outcomes. Adjusted models were fitted using cycles with available data for the corresponding outcome and covariates.

To evaluate whether the observed associations depended on a single DFI cutoff value, additional analyses were performed using clinically interpretable DFI categories and alternative DFI thresholds. DFI categories were defined as ≤15%, 15%–20%, 20%–30%, and >30%. Threshold analyses were performed using DFI cutoffs of 15%, 20%, 25%, and 30%, with the lower DFI group used as the reference group for each threshold.

As an additional analysis, fresh embryo-transfer status was summarized according to DFI category using linked treatment-cycle records. Transfer-day characteristics were summarized among fresh-transfer cycles with available transfer-day information.

Missingness of key clinical and laboratory variables was summarized descriptively. All statistical tests were two sided, and P values < 0.05 were considered statistically significant.

## 3 Results

### 3.1 Study cohort and baseline characteristics

A total of 2,429 fresh oocyte-retrieval treatment-cycle records between August 2021 and December 2025 were identified from the cycle-search database. After sequential filtering, 2,427 fresh IVF oocyte-retrieval treatment cycles, 2064 cycles labeled as conventional IVF, 1872 cycles without ICSI or rescue ICSI counts, and 1,665 cycles with valid sperm DFI and core embryological records required for the stage-specific analyses remained. After retaining only the first eligible conventional IVF cycle for each female patient, 1,423 cycles were included in the final analytical cohort. The cohort selection process and sperm DFI distribution are shown in Figure 1.

**FIGURE 1:**
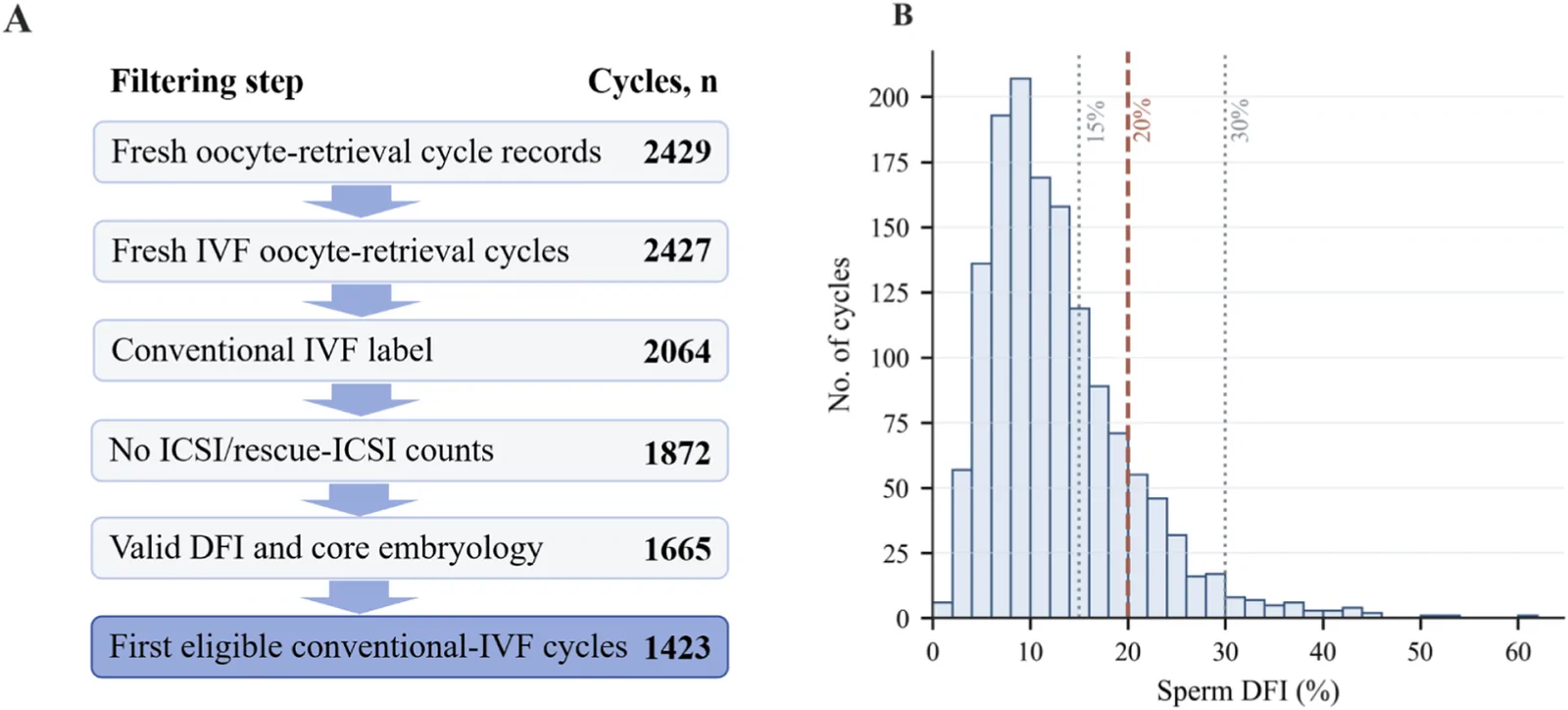
Cohort selection and sperm DFI distribution in first eligible conventional IVF cycles. **(A)** Cohort selection process. **(B)** Distribution of sperm DFI.

Using the primary DFI threshold of 20%, 1,218 cycles were classified into the lower DFI group and 205 cycles into the elevated DFI group. Baseline characteristics according to DFI status are shown in Table 1. Female age was higher in the elevated DFI group than in the lower DFI group [33.00 (30.00–37.00) vs. 31.00 (28.00–35.00) years, P < 0.001]. Male age showed a similar difference [34.00 (31.00–39.00) vs. 32.00 (29.00–35.00) years, P < 0.001].

**TABLE 1:** Baseline characteristics of the analytical cohort according to sperm DFI status.

Characteristics	Lower DFI ≤20%	Elevated DFI >20%	P-value
No. Of cycles	1,218	205	​
Female age, years	31.00 [28.00, 35.00]	33.00 [30.00, 37.00]	<0.001
Male age, years	32.00 [29.00, 35.00]	34.00 [31.00, 39.00]	<0.001
Female BMI, kg/m^2^	22.40 [20.30, 24.90]	23.30 [20.90, 25.40]	0.011
Basal FSH, IU/L	6.50 [5.60, 7.60]	6.60 [5.60, 7.70]	0.756
Progressive sperm motility, %	61.80 [47.00, 72.00]	52.00 [41.00, 65.22]	<0.001
Normal sperm morphology, %	5.00 [3.40, 7.20]	4.80 [2.90, 6.65]	0.01
Oocytes inseminated, n	10.00 [6.00, 15.00]	10.00 [5.00, 14.00]	0.05
Sperm DFI, %	10.00 [7.01, 13.69]	24.30 [22.00, 29.02]	<0.001

Female BMI was slightly higher in the elevated DFI group [23.30 (20.90–25.40) vs. 22.40 (20.30–24.90) kg/m^2^, P = 0.011], whereas basal FSH was comparable between groups [6.60 (5.60–7.70) vs. 6.50 (5.60–7.60) IU/L, P = 0.756]. Progressive sperm motility was lower in the elevated DFI group [52.00% (41.00–65.22) vs. 61.80% (47.00–72.00), P < 0.001], and normal sperm morphology was also slightly lower [4.80% (2.90–6.65) vs. 5.00% (3.40–7.20), P = 0.010]. The number of oocytes inseminated was similar between groups [10.00 (5.00–14.00) vs. 10.00 (6.00–15.00), P = 0.050]. As expected, sperm DFI was higher in the elevated DFI group [24.30% (22.00–29.02) vs. 10.00% (7.01–13.69), P < 0.001].

### 3.2 Embryological transition outcomes according to sperm DFI status

Embryological transition outcomes according to sperm DFI status are shown in Figure 2. The oocyte-to-2PN transition was similar between the two groups. In the lower DFI group, 8,401 of 13,193 oocytes formed 2 P N embryos, corresponding to a normal fertilization rate of 63.7%. In the elevated DFI group, 1,257 of 2018 oocytes formed 2 P N embryos, with a rate of 62.3%.

**FIGURE 2:**
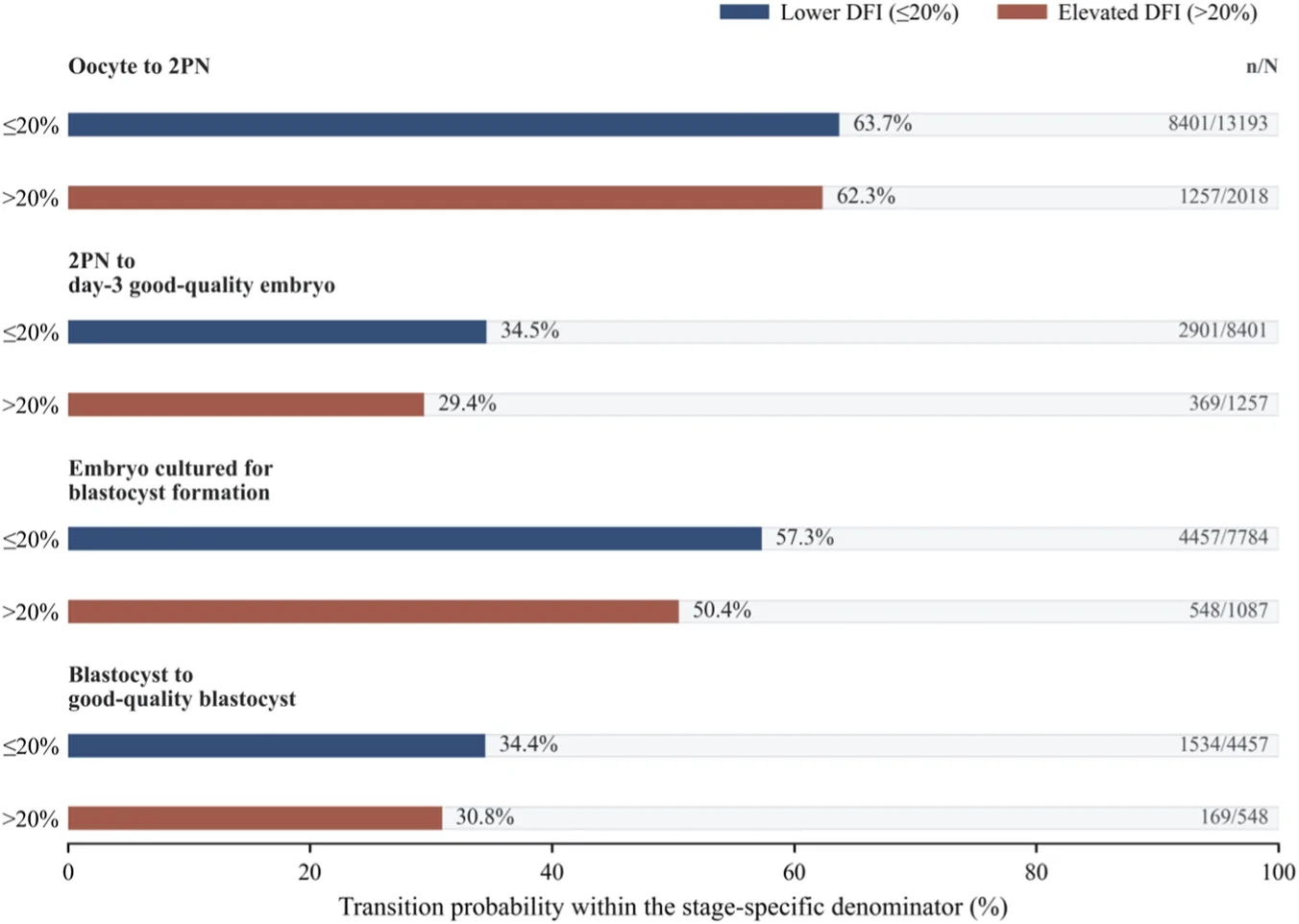
Embryological transition outcomes according to sperm DFI status.

After normal fertilization, the transition pattern began to separate between groups. The 2 P N-to-day-3 good-quality embryo formation rate was 34.5% in the lower DFI group and 29.4% in the elevated DFI group, corresponding to 2,901 of 8,401 and 369 of 1,257 normally fertilized 2 P N embryos, respectively.

For embryos cultured for blastocyst development, blastocyst formation was also lower in the elevated DFI group. Blastocysts formed from 4,457 of 7,784 cultured embryos in the lower DFI group and from 548 of 1,087 cultured embryos in the elevated DFI group, giving rates of 57.3% and 50.4%, respectively.

Among formed blastocysts, the proportion classified as good quality was 34.4% in the lower DFI group and 30.8% in the elevated DFI group. The absolute difference at this stage was smaller than that observed for 2 P N-to-day-3 good-quality embryo formation and blastocyst formation.

Overall, the descriptive data showed little difference between groups at the fertilization step, whereas lower transition rates were observed in the elevated DFI group during subsequent embryo development.

### 3.3 Multivariable associations between elevated sperm DFI and embryological transitions

Multivariable binomial regression results are shown in Figure 3. After adjustment for female age, male age, female BMI, and basal FSH, elevated sperm DFI was not associated with the oocyte-to-2PN transition. The adjusted OR was 0.96 (95% CI, 0.84–1.10; P = 0.523).

**FIGURE 3:**
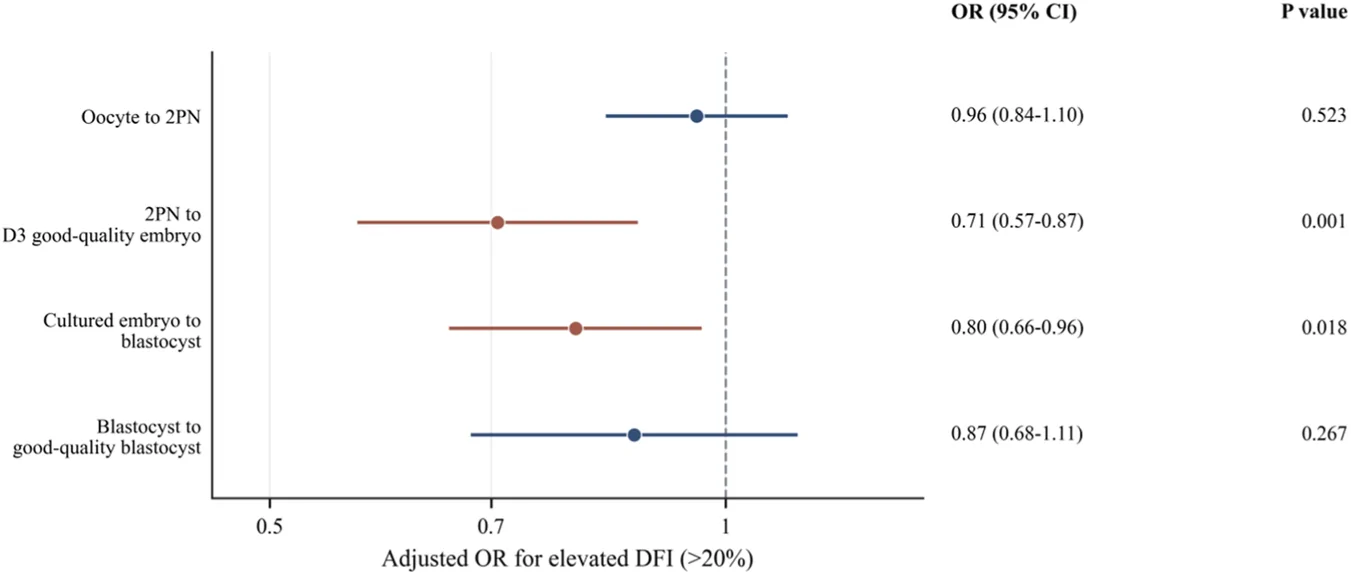
Adjusted associations between elevated sperm DFI and embryological transition outcomes.

For embryo development after fertilization, elevated DFI was associated with lower odds of day-3 good-quality embryo formation. The adjusted OR for 2 P N-to-day-3 good-quality embryo formation was 0.71 (95% CI, 0.57–0.87; P = 0.001). Elevated DFI was also associated with reduced blastocyst formation among embryos cultured for blastocyst development (57.3% vs. 50.4%; adjusted OR, 0.80; 95% CI, 0.66–0.96; P = 0.018).

For good-quality blastocyst formation among formed blastocysts, the adjusted OR was 0.87 (95% CI, 0.68–1.11; P = 0.267).

Taken together, elevated DFI showed little evidence of an association with oocyte-to-2PN formation, whereas inverse associations were observed with 2 P N-to-day-3 good-quality embryo formation and blastocyst formation among embryos entering extended culture.

To evaluate whether the primary findings were influenced by conventional semen quality and ovarian response, an expanded adjustment model was further fitted by additionally including normal sperm morphology and the number of oocytes inseminated. The overall pattern was consistent with the primary multivariable analysis. Elevated sperm DFI remained associated with reduced 2 P N-to-day-3 good-quality embryo formation and reduced blastocyst formation among embryos cultured for blastocyst development. For good-quality blastocyst formation among formed blastocysts, the expanded model likewise yielded a point estimate below 1.0 with a confidence interval that included the null, as shown in Table 2.

**TABLE 2:** Expanded adjustment analysis for embryological transition outcomes.

Embryological transition	Adjusted OR	95% CI	P Value	No. Of cycles
Oocyte to 2 P N	0.96	0.84 to 1.10	0.590	1,303
2 P N to day-3 good-quality embryo	0.70	0.57 to 0.87	0.001	1,275
Cultured embryo to blastocyst	0.81	0.67 to 0.98	0.033	1,150
Blastocyst to good-quality blastocyst	0.87	0.68 to 1.12	0.287	984

### 3.4 DFI category and threshold analyses

The category analysis is shown in Figure 4. Across the four DFI categories, the oocyte-to-2PN transition remained relatively stable, ranging from 59.6% to 64.2%. The rate was 64.2% in cycles with DFI ≤15%, 61.1% in cycles with DFI between 15% and 20%, 62.8% in cycles with DFI between 20% and 30%, and 59.6% in cycles with DFI >30%.

**FIGURE 4:**
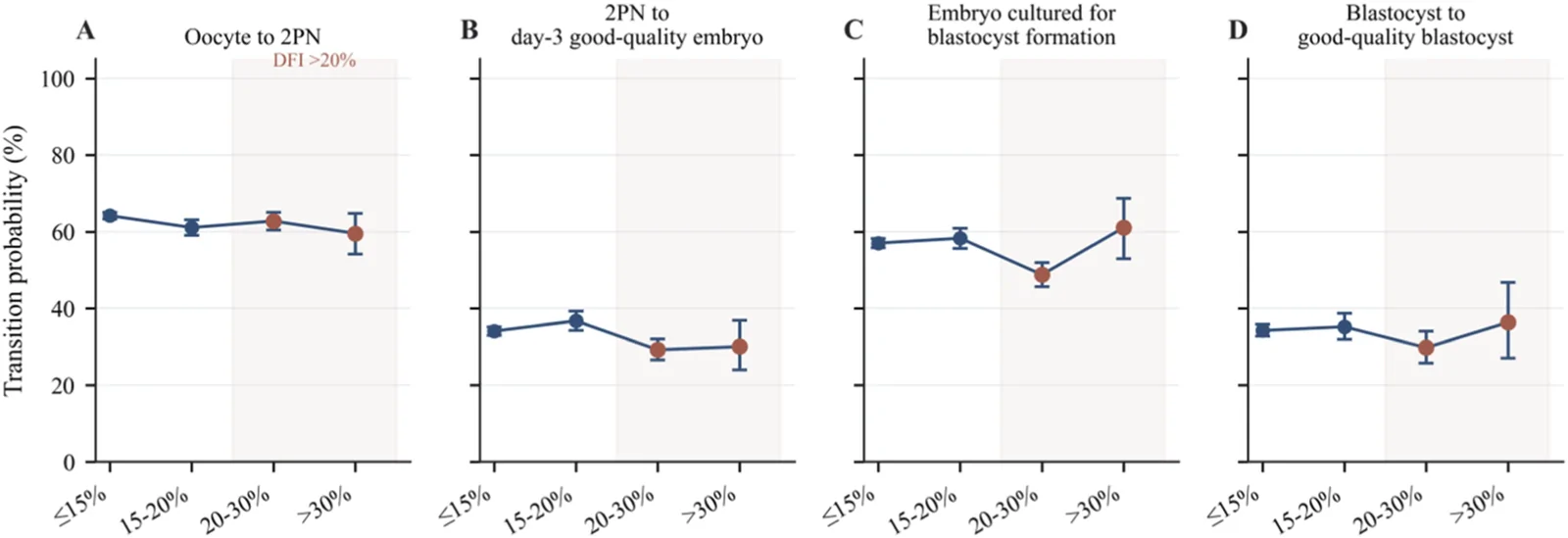
Embryological outcomes across clinically interpretable sperm DFI categories. **(A)** Oocyte-to-2PN formation. **(B)** 2PN-to-day-3 good-quality embryo formation. **(C)** Blastocyst formation among embryos cultured for blastocyst development. **(D)** Blastocyst-to-good-quality blastocyst formation.

For post-fertilization development, the 2 P N-to-day-3 good-quality embryo rate was 34.1% in the DFI ≤15% group and 36.7% in the DFI 15%–20% group, compared with 29.2% in the DFI 20%–30% group and 30.1% in the DFI >30% group. Blastocyst formation rates were 57.0%, 58.3%, 48.8%, and 61.1% across the same four categories, respectively. The DFI >30% category comprised 43 cycles and 144 embryos entering extended culture.

Threshold analyses for the two main post-fertilization outcomes are shown in Figure 5. For 2 P N-to-day-3 good-quality embryo formation, the association was not evident at the 15% threshold (adjusted OR, 0.93; 95% CI, 0.79–1.09; P = 0.365). The association was clearest at the 20% threshold (adjusted OR, 0.71; 95% CI, 0.57–0.87; P = 0.001). At higher thresholds, the estimates remained below 1.0 but were less stable: the adjusted OR was 0.69 for DFI >25% (95% CI, 0.49–0.96; P = 0.028) and 0.75 for DFI >30% (95% CI, 0.51–1.10; P = 0.144).

**FIGURE 5:**
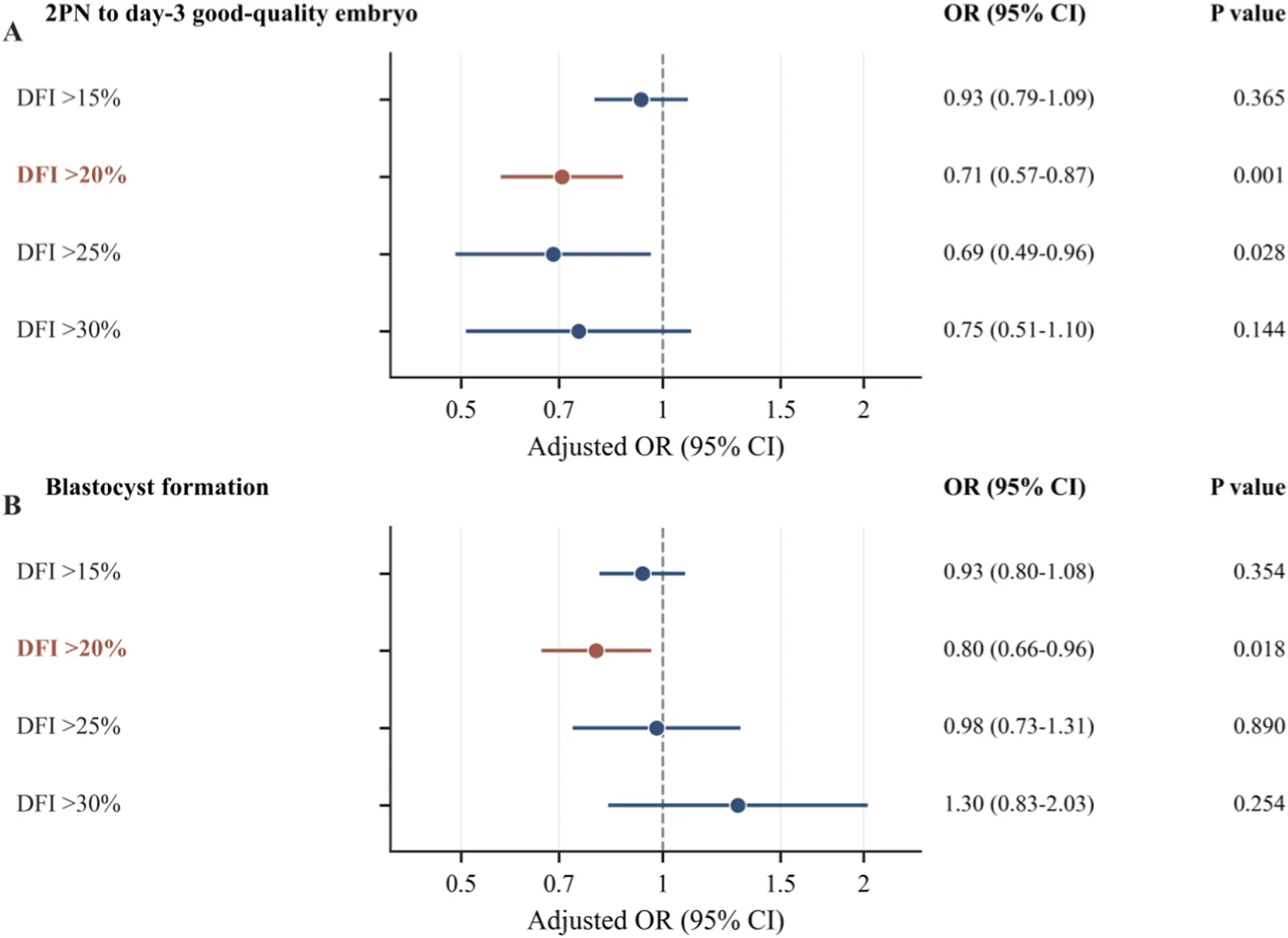
Threshold analyses for the association between sperm DFI and post-fertilization embryological outcomes. **(A)** 2PN-to-day-3 good-quality embryo formation. **(B)** Blastocyst formation among embryos cultured for blastocyst development.

For blastocyst formation, the adjusted ORs were 0.93 for DFI >15% (95% CI, 0.80–1.08; P = 0.354), 0.80 for DFI >20% (95% CI, 0.66–0.96; P = 0.018), 0.98 for DFI >25% (95% CI, 0.73–1.31; P = 0.890), and 1.30 for DFI >30% (95% CI, 0.83–2.03; P = 0.254). Consistent with the category analysis, inverse associations for the two main post-fertilization outcomes were most evident at the 20% threshold, whereas estimates at higher thresholds became less stable as the number of cycles in the elevated-DFI groups decreased.

### 3.5 Fresh embryo-transfer characteristics

Fresh embryo transfer was performed in 386 of 1,218 cycles (31.7%) in the lower-DFI group and in 81 of 205 cycles (39.5%) in the elevated-DFI group. Transfer-day information was available for 466 of the 467 fresh-transfer cycles. No day-2 transfers were identified; 261 transfers (56.0%) were performed on day 3 and 205 (44.0%) on day 5. The distribution of transfer day did not differ significantly between the lower- and elevated-DFI groups (P = 0.254). Detailed fresh embryo-transfer characteristics are presented in Supplementary Table S1.

## 4 Discussion

In this retrospective cohort of 1,423 first eligible conventional IVF cycles, elevated sperm DFI was not associated with normal fertilization but was associated with reduced post-fertilization developmental efficiency. The association was most evident for the transition from normally fertilized 2 P N embryos to day-3 good-quality embryos and was also observed for blastocyst formation among embryos cultured for blastocyst development.

The relationship between sperm DNA fragmentation and ART outcomes has been debated for many years, with individual studies and evidence syntheses reaching variable conclusions regarding fertilization, embryo development, and downstream reproductive outcomes (Bareh et al., 2016; Ribas-Maynou et al., 2021; Maghraby et al., 2024; Dabiri et al., 2026). Earlier studies likewise reported heterogeneous associations with specific embryological endpoints, including fertilization, cleavage-stage development, embryo kinetics, and blastocyst formation (Virro et al., 2004; Machałowski et al., 2025; Alvarez Sedó et al., 2017; Esbert et al., 2018). Recent studies have continued to show substantial heterogeneity across developmental endpoints and ART settings. Jiang et al. (2025), in a large cohort including IVF and ICSI cycles, found no consistent association between DFI and embryological parameters when the two fertilization methods were analyzed separately, although an inverse association with fertilization was observed in the pooled ART analysis. In an ICSI cohort, Machałowski et al. (2025) reported lower fertilization and poorer day-5 blastocyst quality with increasing sperm DNA fragmentation, whereas the association with day-3 embryo quality was less certain. In conventional IVF, Mori et al. (2025) reported inverse associations between DFI and blastocyst formation and quality, although the study was exploratory and included a relatively small number of men. Chuang et al. (2026) subsequently observed lower fertilization and cleavage rates in IVF cycles with SDF ≥20%. By contrast, Wei et al. (2026), in a large real-world ART cohort, found an association between high DFI and lower 2 P N fertilization but no significant association with top-quality or transferable embryo outcomes.

The DFI assay used in the present study was based on acid-induced DNA denaturation followed by differential acridine-orange fluorescence. This analytical principle is related to other AO-based flow-cytometric approaches, including the sperm chromatin structure assay (SCSA), although the present commercial assay should not be regarded as identical to the standardized SCSA protocol. In an earlier SCSA study, Virro et al. (2004) found no significant difference in fertilization between high- and low-DFI groups, whereas higher DFI was associated with a greater risk of poor blastocyst development. This pattern is broadly consistent with the stage-specific separation observed in the present conventional-IVF cohort. More recently, Jiang et al. (2025), using an acridine orange test with flow cytometry, found no significant association between DFI and embryological parameters when IVF and ICSI cycles were analyzed separately, although DFI was inversely associated with fertilization when the ART cycles were pooled. Taken together, these findings also illustrate that associations with DFI may vary according to study population, fertilization method, assay implementation, DFI threshold, and the denominator used to define individual embryological outcomes. Because AO-based assays quantify differential fluorescence after acid-induced DNA denaturation rather than directly labeling DNA strand-break termini, DFI values should be interpreted in the context of the specific analytical protocol used.

Our finding that elevated DFI was not significantly associated with normal fertilization is biologically plausible. Fertilization depends on several sperm functions, including motility, capacitation, acrosome reaction, sperm-oocyte binding, and oocyte activation. These processes may remain adequate even when sperm DNA integrity is reduced. In addition, early zygotic events are largely supported by oocyte cytoplasmic factors and maternally stored transcripts (Newman et al., 2022). Oocytes also possess DNA repair capacity, which may partially compensate for paternal DNA damage during the earliest stages after fertilization (González-Marín et al., 2012). Therefore, sperm DNA fragmentation may not necessarily prevent the formation of 2 P N embryos. This interpretation is also consistent with the concept of a late paternal effect, in which sperm DNA damage affects embryo development after fertilization rather than the fertilization event itself (Pérez-Cerezales et al., 2010; Tesarik et al., 2004).

The association between elevated DFI and lower 2 P N-to-day-3 good-quality embryo formation supports the possibility of a post-fertilization paternal effect. Human embryonic genome activation occurs mainly between the four-cell and eight-cell stages, and transcriptional activity increases during cleavage-stage development (Xue et al., 2013; Yan et al., 2013). As embryonic development progresses, paternal genome integrity may become more relevant for sustaining normal cell division, maintaining genomic stability, and supporting subsequent embryo development. In the present study, the difference between DFI groups became apparent during the transition from normally fertilized 2 P N embryos to day-3 good-quality embryos. This finding suggests that early post-fertilization developmental competence may be more sensitive to sperm DNA fragmentation than the fertilization step itself.

Blastocyst formation showed a concordant but differently interpretable pattern. Among embryos entering extended culture, elevated DFI was associated with a lower probability of blastocyst formation. This outcome is denominator-dependent because blastocyst status is observed only for embryos undergoing extended culture. The observed association therefore describes blastocyst formation conditional on extended culture and is distinct from an oocyte-based or 2 P N-based blastocyst yield. For this reason, the blastocyst endpoint was considered complementary to the 2 P N-to-day-3 developmental transition.

Among formed blastocysts, good-quality blastocyst formation was 34.4% in the lower-DFI group and 30.8% in the elevated-DFI group, with an adjusted OR of 0.87 (95% CI, 0.68–1.11; P = 0.267). Because this analysis was conditional on blastocyst formation, it addresses morphological quality among blastocysts that had reached this developmental stage (Capalbo et al., 2014; Balaban et al., 2011).

The clinical implications of these findings should be interpreted in the context of embryological development rather than treatment selection alone. The results do not suggest that sperm DFI should be used as a stand alone diagnostic marker or as the sole basis for choosing a fertilization strategy in conventional IVF cycles (Pfeifer et al., 2013; Aitken and Bakos, 2021). Instead, elevated DFI may provide additional information about embryo development after fertilization. For couples undergoing conventional IVF, elevated DFI may not necessarily indicate reduced normal fertilization, but it may be associated with lower developmental efficiency after fertilization. This distinction is clinically relevant because it separates fertilization potential from subsequent embryo developmental competence.

This study has several strengths. First, the analysis was restricted to the first eligible conventional IVF cycle for each female patient, reducing the influence of repeated treatment cycles. Second, cycles involving ICSI, rescue ICSI, or mixed insemination were excluded, allowing the analysis to focus on conventional IVF cycles in which fertilization occurred without micromanipulation. Third, embryological development was analyzed using stage-specific denominators rather than broad summary percentages. This approach avoided combining oocyte-based, 2 P N-based, culture-based, and blastocyst-based outcomes in a single calculation. Fourth, the analysis separated normal fertilization, 2 P N-to-day-3 good-quality embryo formation, blastocyst formation, and good-quality blastocyst formation, which helped identify where the association with elevated DFI was most apparent.

Several limitations should be considered. This was a single-center retrospective study based on routinely collected laboratory data, and information on some paternal lifestyle factors was unavailable. DFI was measured using a single AO-based acid-denaturation assay. Blastocyst outcomes were conditional on entry into extended culture, and PGT-A was not performed in this cohort. The present analysis focused on pre-transfer embryological development; cumulative pregnancy and live-birth outcomes were not evaluated.

## 5 Conclusion

In first eligible conventional IVF cycles, elevated sperm DFI was associated with developmental attrition after normal fertilization rather than with impaired fertilization itself. The association emerged during cleavage-stage development, with reduced 2 P N-to-day-3 good-quality embryo formation, and was also evident for blastocyst formation among embryos entering extended culture. These findings support a stage-specific interpretation of sperm DNA fragmentation in conventional IVF and suggest that paternal DNA integrity is more closely related to post-fertilization developmental competence than to fertilization potential.

## Data Availability

The raw data supporting the conclusions of this article will be made available by the authors, without undue reservation.
