# Supplementary material for: Elevated sperm DNA fragmentation is associated with embryonic developmental attrition after fertilization in conventional IVF cycles
Source: Front Cell Dev Biol. 2026 Sep 21;14:1925444. doi: 10.3389/fcell.2026.1925444 (PMC13635119; doi:10.3389/fcell.2026.1925444)
Supplement: Supplementary file 1 [file Table1.docx]

Supplementary Table S1. Fresh embryo-transfer characteristics according to sperm DFI status

| **Characteristic** | **DFI ≤20%** | **DFI >20%** | **P value** |
| --- | --- | --- | --- |
| Analytical cycles, n | 1218 | 205 | — |
| Fresh embryo transfer, n (%) | 386 (31.7) | 81 (39.5) | 0.027 |
| No fresh embryo transfer, n (%) | 832 (68.3) | 124 (60.5) |  |
| Fresh-transfer cycles with recorded transfer day, n | 385 | 81 | — |
| Day 2, n (%) | 0 (0.0) | 0 (0.0) | — |
| Day 3, n (%) | 211 (54.8) | 50 (61.7) | 0.254 |
| Day 5, n (%) | 174 (45.2) | 31 (38.3) |  |

Fresh embryo-transfer percentages were calculated using all analytical cycles within each DFI category as the denominator. Transfer-day percentages were calculated among fresh-transfer cycles with recorded transfer-day information. Transfer day was unavailable for one fresh-transfer cycle in the DFI ≤20% group. P values were calculated using Pearson’s chi-square test.

**Supplementary Methods**

1. Routine semen collection and assessment

Semen samples were collected and analyzed according to the standard operating procedures of the reproductive laboratory. Male partners were instructed to maintain sexual abstinence for 2–7 days before sample collection. Samples were obtained by masturbation and analyzed after liquefaction. Routine semen parameters, including semen volume, sperm concentration, progressive motility, and morphology, were assessed according to laboratory procedures based on the World Health Organization laboratory manual for the examination and processing of human semen.

2. Ovarian stimulation and oocyte retrieval

Controlled ovarian stimulation was performed using individualized protocols according to female age, ovarian reserve, previous ovarian response, and physician judgment. Oocyte retrieval was performed approximately 36.5 h after triggering under transvaginal ultrasound guidance. Fertilization and embryo culture were subsequently performed according to the standard operating procedures of the reproductive laboratory.

3. Cleavage-stage embryo assessment

Cleavage-stage embryos were evaluated on days 2 and 3 according to blastomere number, blastomere size and symmetry, cytoplasmic appearance, and the degree of fragmentation. Under the laboratory grading system, grade I embryos had evenly sized blastomeres, regular morphology, homogeneous and clear cytoplasm, and ≤10% fragmentation. Grade II embryos showed slightly uneven blastomeres or mildly irregular morphology, mild changes in cytoplasmic refractility, and ≤20% fragmentation. Grade III embryos had uneven blastomeres, irregular morphology, coarse cytoplasmic granularity, and <50% fragmentation. Grade IV embryos showed marked blastomere unevenness, severely irregular morphology, cytoplasmic aggregation or heterogeneous granularity, and ≥50% fragmentation. Grade V embryos were considered nonviable and showed degenerated, shrunken, or darkened blastomeres.

Day-3 good-quality embryos were defined as grade I or grade II embryos. Embryos with 7–10 cells on day 3 were considered developmentally appropriate when the corresponding morphological criteria were met; embryos showing delayed or excessively rapid cleavage were not classified as optimal-quality embryos according to the laboratory assessment criteria.

4. Blastocyst assessment

Blastocysts were evaluated according to the Gardner morphological grading system and the laboratory standard operating procedure. Blastocyst expansion was graded from 1 to 6. Inner cell mass and trophectoderm morphology were each graded as A, B, or C according to cell number, compactness, and epithelial organization. Good-quality blastocysts were defined as day-5 blastocysts graded 3BB or higher and day-6 blastocysts graded 4BB or higher.
